# Peripheral blood mononuclear cells (PBMC) microbiome is not affected by colon microbiota in healthy goats

**DOI:** 10.1186/s42523-021-00091-7

**Published:** 2021-04-14

**Authors:** Ainize Peña-Cearra, Alejandro Belanche, Monika Gonzalez-Lopez, José Luis Lavín, Miguel Ángel Pascual-Itoiz, Elisabeth Jiménez, Héctor Rodríguez, Ana Mª. Aransay, Juan Anguita, David R. Yáñez-Ruiz, Leticia Abecia

**Affiliations:** 1 CIC bioGUNE, Bizkaia Science and Technology Park, bld 801 A, 48160 Derio, Bizkaia Spain; 2 Departamento de Inmunología, Microbiología y Parasitología, Facultad de Medicina y Enfermería, Universidad del País Vasco/Euskal Herriko Unibertsitatea (UPV/EHU), Apartado 699, 48080 Bilbao, Spain; 3 Estación Experimental del Zaidín (CSIC), 18008 Granada, Spain; 4 Present Address: NEIKER Instituto Vasco de Investigación y Desarrollo Agrario, Parque Tecnológico Bizkaia Ed. 812, 48160 Derio, Spain; 5 CIBERehd, ISCIII, Madrid, Spain; 6 Ikerbasque, Basque Foundation for Science, Bilbao, Bizkaia Spain

**Keywords:** Blood immune cells, Ruminants, Immunoglobulins, Translocation

## Abstract

**Background:**

The knowledge about blood circulating microbiome and its functional relevance in healthy individuals remains limited. An assessment of changes in the circulating microbiome was performed by sequencing peripheral blood mononuclear cells (PBMC) bacterial DNA from goats supplemented or not in early life with rumen liquid transplantation.

**Results:**

Most of the bacterial DNA associated to PBMC was identified predominantly as *Proteobacteria* (55%) followed by *Firmicutes* (24%), *Bacteroidetes* (11%) and *Actinobacteria* (8%). The predominant genera found in PBMC samples were *Pseudomonas*, *Prevotella, Sphingomonas, Acinetobacter, Corynebacterium* and *Ruminococcus*. Other genera such as *Butyrivibrivio, Bifidobacterium*, *Dorea* and *Coprococcus* were also present in lower proportions. Several species known as blood pathogens or others involved in gut homeostasis such as *Faecalibacterium prausnitzii* were also identified. However, the PBMC microbiome *phylum* composition differed from that in the colon of goats (*P* ≤ 0.001), where *Firmicutes* was the predominant *phylum* (83%). Although, rumen liquid administration in early-life altered bacterial community structure and increased *Tlr5* expression (*P* = 0.020) in colon pointing to higher bacterial translocation, less than 8% of OTUs in colon were also observed in PBMCs.

**Conclusions:**

Data suggest that in physiological conditions, PBMC microbiome differs from and is not affected by colon gut microbiota in small ruminants. Although, further studies with larger number of animals and covering other animal tissues are required, results point to a common circulating bacterial profile on mammals being *phylum Proteobacteria,* and genera *Pseudomonas* and *Prevotella* the most abundants*.* All suggest that PBMC microbiome in healthy ruminants could be implicated in homeostatic condition. This study expands our knowledge about PBMC microbiome contribution to health in farm animals.

**Supplementary Information:**

The online version contains supplementary material available at 10.1186/s42523-021-00091-7.

## Introduction

The circulation is a closed system and the blood in healthy organism was first believed to be a sterile environment [1]. However, the principle of the presence of truly sterile blood in healthy individuals has been challenged, as operationally it does not mean that dormant or non-culturable forms of organisms are absent [2]. Over the last few years, an increasing number of bacteria blood culture isolates have been reported. Therefore the concept of blood microbiome has been emerging although is still under debate. Usually, the presence of a blood microbiome has also been associated with a variety of diseases. Indeed, the etiology of diabetes, cardiovascular disease, hematological disorders and cirrhosis has been ascribed to the translocation of bacteria from the intestinal tract, primarily via the intestinal epithelial mucosa [3–7]. Consequently, the gut microbiome was thought to be the main contributor of the blood microbiome, although the origin of these bacteria is still unknown. Moreover, blood microbial DNA (plasma derived, cell free microbial nucleic acids) was recently proposed as a potential tool to discriminate between numerous types of cancer and healthy individuals [8] providing more evidences of particular microbial DNA in relation to health status.

The first evidence of microbial presence in the blood was found nearly 20 years ago by Nikkari et al. [9] who reported that healthy blood specimens can contain bacterial *16S rRNA* gene. Detection of *16S rRNA* gene does not confirm the presence of viable microbes and external DNA contamination could account for bacterial DNA found in the hematic samples. Potgieter et al. [10], used transmission electron microscopy analysis to show the presence of bacteria internalized in erythrocytes providing further evidences of a blood microbiome. Later, the presence of comparable bacterial phyla in different studies appears to support the existence of a healthy human blood microbiome [2–4, 11–16]. Then, more research raised a new paradigm, proposing that healthy individuals harbour a rich microbiota in their blood, including known pathogens that can survive in a dormant form in blood and inside red blood cells [17, 18]. However, the distribution of microbial DNA among white blood cells is unknown although could be also relevant in the physiological transport of bacteria in circulation. According to recent studies from our group, symbiotic bacteria *Lactobacillus plantarum* used as probiotic and found in extraintestinal sites such as blood or milk, was able to survive inside healthy human’s monocytes up to 24 h [19]. Thus, it seems possible that bacteria from different origins may translocate into the systemic circulation, but not being detected through standard culture methods. Currently, the use of recently developed tools to minimize contributions of contaminants to microbial signatures could be applied to blood from healthy individuals to identify the presence of bacterial DNA [20–23]. Following specific protocols to control contaminants, Paϊssé et al. [12] showed that a diverse microbiota was present in the blood of healthy human individuals. Accordingly, it seems necessary to ascertain whether this is also a trait of other mammals to better understand the different microbiome and their relationships with health in farm animals. In this study, we aimed to evaluate the peripheral blood mononuclear cells (PBMC) bacterial microbiome in healthy goats. In order to promote substantial changes in the microbial diversity in colon content with potential to translocate into blood cells, two groups of ruminants were used. One group was orally inoculated during early life with rumen liquid from adults and the other without inoculation.

## Material and methods

All management and experimental procedures involving animals were performed by trained personnel according to the Spanish guidelines (RD 53/2013). Experimental protocols were approved by the Ethical Committee for Animal Research (EEZ-CSIC) regional government (09/03/2017).

### Animals and experimental design

Sixteen male goat kids were used in this experiment. All kids were raised with milk-replacer (Sello azul, Lemansa, León, Spain) and randomly distributed into 2 treatments (*n* = 8): one group was orally inoculated with pooled fresh rumen liquid transplantation (RLT) from 4 adult goats whereas the control group (CTL) did not receive inoculation. Details of the inoculation process were previously published [24]. Briefly, rumen inocula were obtained from donor animals fitted with permanent rumen fistula which were fed a diet based on a 70% of concentrate and 30% of forage. Rumen fluid was daily collected, filtered through two layers of muslin and immediately inoculated to the RLT animals by oral drenching of 2.5 ml/animal during week 1 and 5 ml/animal thereafter. Inoculation was daily repeated from birth until 2.5 months of age when they were fully adapted to a solid diet. Both groups were separated from each other to avoid physical contact and potential microbial transfer. Animals were weaned at the age of 7 weeks and experimental groups remained separated throughout the entire experiment. At 6 months of age, blood samples were taken from the jugular vein, animals were slaughtered and colon content was sampled and snap frozen in liquid nitrogen. Samples of the colon tissue were washed immediately after collection with 0.01 M phosphate-buffered saline (PBS) buffer (pH 6.8). The samples of washed tissue were then transferred to RNA later solution (Qiagen Ltd., West Sussex, UK) and stored at − 80 °C until further analysis.

### Gene expression analysis in colonic tissue

Total RNA was extracted from colon tissue. They were homogenized with 0.9 mm stainless steel bead and 1 ml of TRIzol Reagent (Invitrogen) using a bullet Blender homogenizer as previously described [25]. RNA integrity number was measured using Bioanalyzer 2100 (Agilent Technologies, Santa Clara, CA). The extracted total RNA was reversed transcribed using M-MLV reverse transcriptase (Thermo) and the yielded cDNA was used as template for real time quantitative PCR (RTqPCR) analysis to evaluate the expression of *Tlr2, 4, 5* and *9, β-defensin* and *peptidoglycan recognition protein 1* (*Pglrp1*) genes. RTqPCR amplification and detection was performed on optical grade 384-well plates in a ViiA7 Real-Time PCR System (Thermo Fisher Scientific, Waltham, MA, USA) with PerfeCTa qPCR Tough Mix (Quantabio, Beverly, MA, USA). Specific primers at their annealing temperature were used as previously reported [25]. To normalize mRNA expression, *β-actin* was used as housekeeping gene. The mRNA relative quantification was calculated using the ΔΔCt method. PCR efficiency was always between 90 and 110%, and a negative control was run for each set of primers.

### Samples preparation and DNA extraction

Blood samples were collected into EDTA coated vacutainer. Peripheral blood mononuclear cells (PBMC: lymphocytes and monocytes) isolation was performance following ficoll density gradient method based on the principle of differential migration of blood cells through the media during the centrifugation (400×g for 40 min, at room temperature and without break). Cells were preserved in Trizol. The DNA extraction from PBMC was performed to minimize any risk of contamination according to TRIzol reagent protocol (15,596,026.PPS) in a laminar air-flow hood cleaned with 70% ethanol, and UV-irradiated for 20 min before execution of sample processsing. The quantity of extracted DNA was measured by Qubit (Thermofisher Scientific). DNA extracts were stored at − 20 °C until further processing. An empty vial was used as a template-free “negative blank” into which each reagent (new and filtrated) used was added and further processed with the same DNA extraction protocol and amplicon production method as the experimental PBMC samples, and sequenced on the same run to ensure the absence of artifacts such as bacterial DNA contaminants from reagents or nonspecific amplification of eukaryotic DNA.

In order to understand the translocation mechanism in ruminants, colon content was also analyzed. After freeze drying and homogenizing, DNA from colon content was extracted using QIAamp DNA Stool Mini Kit following manufacturer’s protocol and employing the 95 °C heating option. Concentrations of total bacteria in colon content were determined by quantitative PCR as previously described [25]. Primer sets used were as follows: forward GTGSTGCAYGGYTGTCGTCA and reverse ACGTCRTCCMCACCTTCCTC [26].

### *16S rRNA* gene sequencing and data processing

Microbial community composition of samples taken from PBMC and colon was determined using amplicon sequencing of the *16S rRNA* gene of experimental groups of kid (*n* = 3 for CTR and *n* = 4 RFT). Both, PBMCs and colon content sequencing libraries were prepared by an adapted procedure, based on the standard protocol “16S Metagenomic Sequencing Library Preparation from Illumina” (Part # 15044223 Rev. A). In this protocol, a first PCR to amplify template DNA using region of interest-specific primers with overhang adapters attached was carried out. Then, to add dual indexes and Illumina sequencing adapters a second PCR was performed using the Nextera XT Index Kit. Adaptation of this methodology consists of the substitution of the sequences targeting the amplification of the V3-V4 hypervariable region of the *16S rRNA* gene proposed in this protocol by more specific ones (5′-TCCTACGGGAGGCAGCAGT-3′ and 5′-GGACTACCAGGGTATCTAATCCTGTT-3′ [27]). These sequences have been reported to have high sensitivity (targeting 95% of the bacterial sequences found in the Ribosomal Database Project) and 100% specificity (no eukaryotic, mitochondrial, or Archaea DNA targeted) [12]. Colon content libraries were obtained from 12.5 ng of DNA (as recommended) and PBMCs ones from 125 ng of DNA. Region of interest was amplified by PCR (25 cycles) and performed in 25 μl, containing 1x of KAPA HiFi HotStart ReadyMix (Roche Molecular Systems, Inc., Cat.# KK2601), 0.2 μM of specific primers and the mentioned quantities of genomic DNA. To verify the correct amplicon size, 1 μl of PCR product was visualized on a Bioanalyzer High Sensitivity DNA chip (Agilent Technologies, Cat. # 5067–4626) and after purification with AMPure beads (Beckman Coulter, Cat.# A63881), PCR product was eluted in 52.5 μl of elution buffer. Then, 5 μl of cleaned DNA were used in the second PCR (8 cycles), where Nextera-XT adapters (Illumina Inc., Cat.# (FC-131-1001 or FC-131-1002) with dual indexes were added. The second PCR was performed in 50 μl, containing 1x of KAPA HiFi HotStart ReadyMix (Roche Molecular Systems, Inc., Cat.# KK2601), 5 μl of each Illumina Inc.’s adapter and the 5 μl of the purified first PCR product. To verify the performance of the second PCR, 1 μl of each reaction was visualized on a Bioanalyzer High Sensitivity DNA chip. Finally, libraries were cleaned up using AMPure beads, eluted in 25 μl of water, and then quantified using Qubit dsDNA HS DNA Kit (Thermo Fisher Scientific, Cat. # Q32854). The amplicons sequencing was performed on a MiSeq (Illumina Inc.) platform following a standard protocol for paired-end reads of 300 nucleotides.

Quality control of the reads was carried out using FASTQC software (http://www.bioinformatics.babraham.ac.uk/projects/fastqc/). Reads were filtered from the adapter sequences and their quality score using trim_galore software (http://www.bioinformatics.babraham.ac.uk/projects/trim_galore/) and only are retained those with at least 20 phred quality score. Trimmed sequences were merged via FLASH [28] to create amplicons of approximately 460 bp. Amplicons were analysed using QIIME: Quantitative Insights Into Microbial Ecology software package [29]. Sequences were clustered as operational taxonomic units (OTUs) of 97% similarity using uclust [30]. Taxonomic assignment of sequences was performed against the Greengenes database (version 13–8) [31]. The significances of grouping in the PCA plots were tested by analysis of similarity (ANOSIM) with 999 permutations using vegan R-package [32, 33]. The sequences obtained in this study were deposited in the European Nucleotide Archive (ENA) under the project number PRJEB39180.

Then, the relative abundance of each OTU present in the negative-blank was removed from each PBMC library results using R programming language (https://stackoverflow.com/questions/51514356/how-to-subtract-values-of-a-first-column-from-all-columns-by-function-in-r). In the absence of further studies, bioinformatic software (PICRUSt2) was used to investigate functional differences between PBMC and gastrointestinal microbial ecosystem. After normalizing the OTU table through Cumulative Sum Squares (CSS), KEGG Pathways were predicted by PICRUSt algorithm (level 3) [34]. Figure represents differential PICRUSt predicted KEGG pathways between bacterial communities found in PBMC samples compare to colon ones detected by STAMP software [35].

### Statistical analysis

**Fig. 1:**
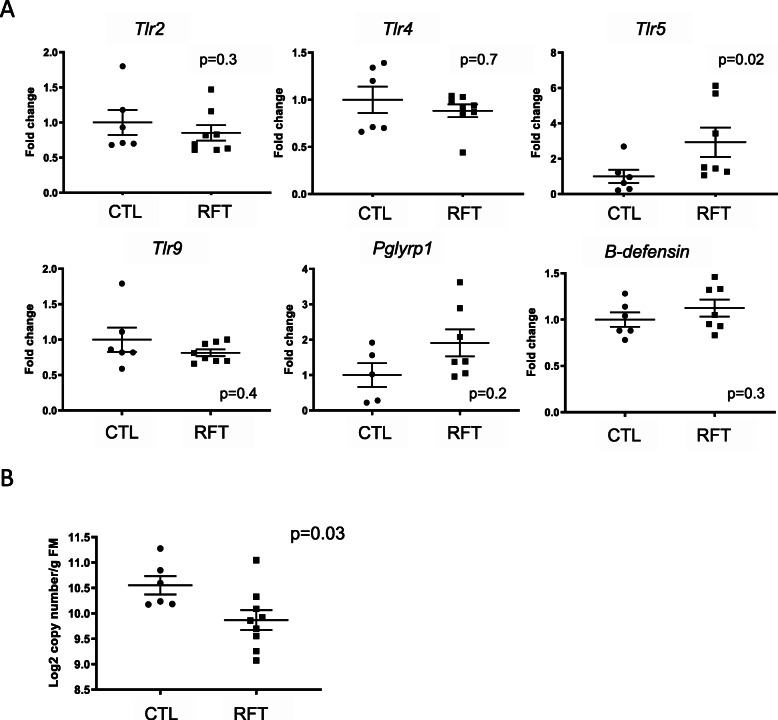
**a** Gene expression level in colon tissue. **b** Total bacterial DNA in colon content determined by qPCR. Experimental groups: CTL (Control) and RLT (rumen liquid transplantation)

Statistical analyses represented in Fig. 1 (nonparametric Mann-Whitney’s tests) were conducted using PRISM (Version 8.05, GraphPad, Inc., La Jolla, CA). The significant fold change of OTU’s was performed using DESeq2 [36]. The STAMP [35] statistical comparison between groups was performed by two-sided Welch’s *t*-test within 95% confidence interval. Spearman’s correlation test was used to assess the relationships among bacterial composition from colon and PBMC. Only OTUs present in both type of samples, in more than 3 goats and which abundance was higher than 20 OTUs per animal were used for the analysis. Only correlations with a value of *P* < 0.05 were considered as significant and were represented.

## Results

Early-life intervention with rumen liquid transplantation did not affect body weight (26 ± 3.21 Kg), colon weight (893.7 ± 97.95 g) or colon pH (6.29 ± 0.54) from the experimental groups.

### Gene expression in colon

The expressions of genes related to host response to microbiota in the colon of goats was measured using RT-qPCR relative to *β-actin* expression using the ΔC_t_ value. Expression of *Pglypr1*, *βdefensin, Tlr2, Tlr4,* and *Tlr9* were not modified by rumen fluid transplant. However, *Tlr5* used by the mucosal immune system of the gut to detect flagellin showed higher expression (*P* = 0.02) in the colon from goats transplanted with rumen fluid early in life (Fig. 1a).

### PBMC and colon content microbial profile

**Fig. 2:**
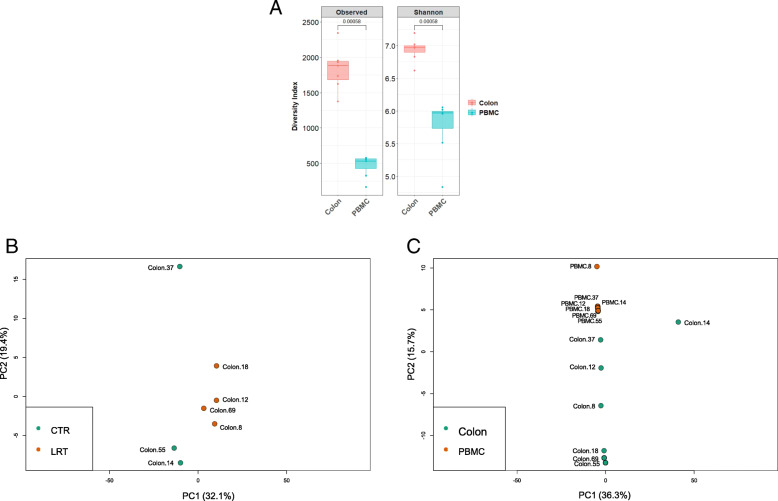
**a** Alpha diversity measurement, observed species and shannon indices in PBMC and colon content samples. **b** PCA plot from control (CTL) and rumen fluid transplantation (RFT) groups (ANOSIM; *p* = 0.028) colon content. **c** PCA plots comparing PBMC and colon microbiome (ANOSIM, *p* = 0.0005) from experimental goats

To evaluate the potential impact of colon microbiota on blood bacterial composition in goats, the taxonomic diversity and profile of the bacterial DNA present in PBMC and colon samples were analysed by high-throughput *16S rRNA* gene amplicon sequencing. A total of 3,387,331 high-quality sequences (reads) were obtained ranging from 193,826 to 222,762 in colon and from 177,386 to 330,387 in PBMC samples. After clustering, 31,421 ± 900 OTUs in colon and 12,904 ± 1042 OTUs in PBMC samples were used for microbial analysis. Good’s coverage was over 99% in all experimental samples. Taxonomic assignment, observed species and Shannon diversity indices (illustrated at the OTU level in Fig. 2a) displayed that colon presents a higher bacterial diversity than PBMC (*P* = 0.001). Then, PCA showed that the supplementation with rumen fluid in early life affected (ANOSIM, *p* = 0.028) ecosystem structure in colon samples (Fig. 2b). In addition, the quantity of bacterial DNA present in the colon was also affected with lower numbers (*P* = 0.03) in goats supplemented with rumen fluid (Fig. 1b). However, in agreement with alpha diversity, when all samples were compared together, PBMC ones were significantly different (ANOSIM, *P ≤* 0.001) from colon samples (Fig. 2c).

**Fig. 3:**
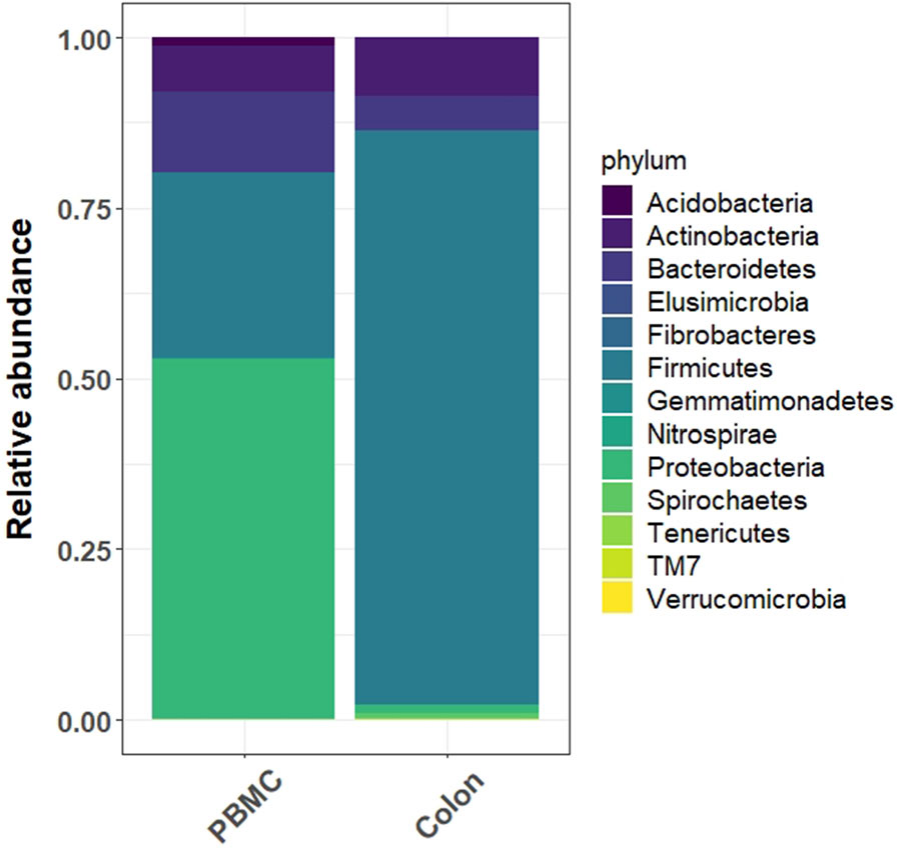
**a** PBMC and colon content *phylum* distribution in experimental goats

**Fig. 4:**
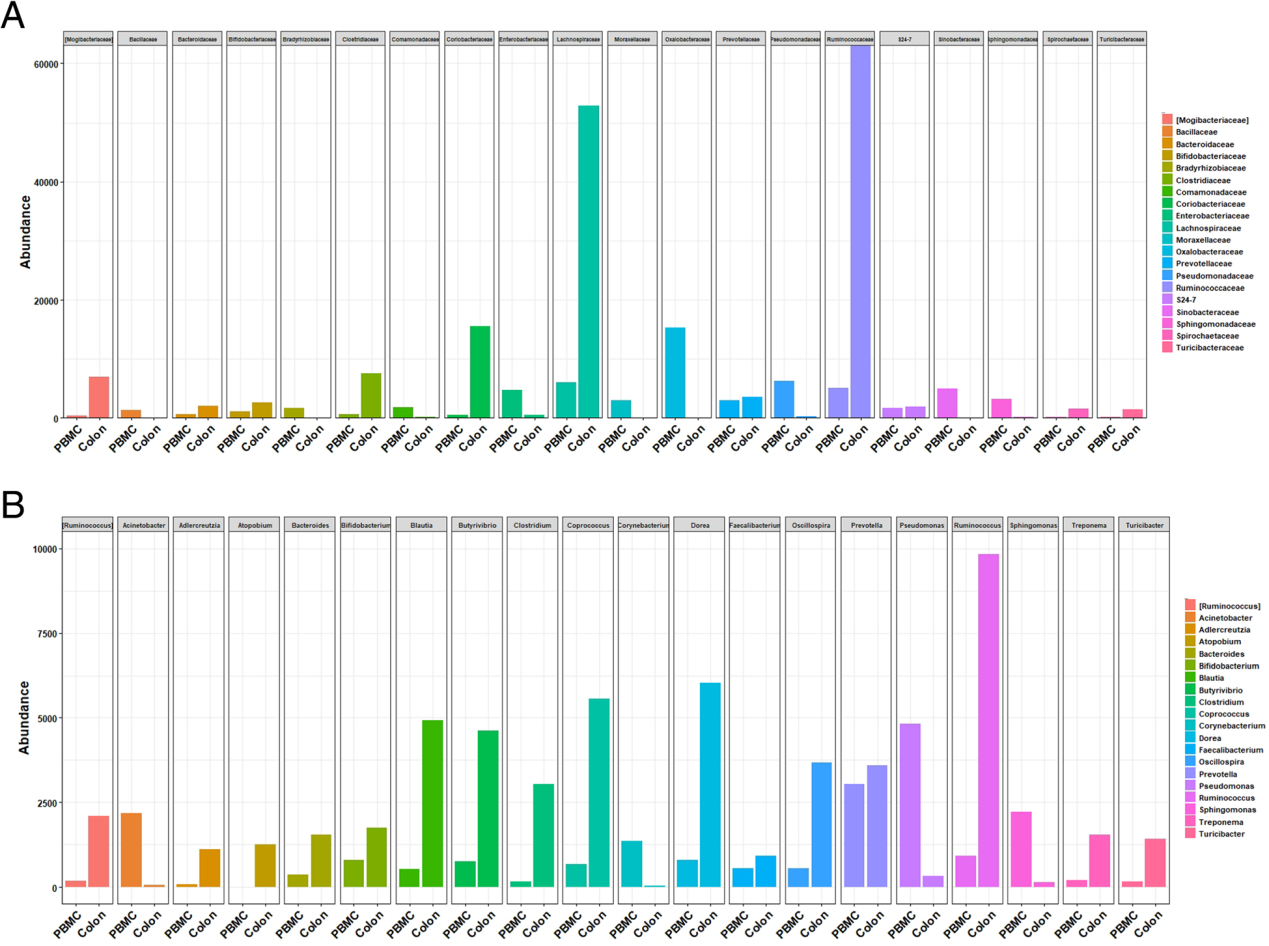
**a** Twenty most abundant families present in PBMC and colon content microbiome. **b** Twenty most abundant genera detected in PBMC and colon content microbial community in healthy goats

The distribution of reads assigned at the *phylum* level (Fig. 3) reveals that the PBMC contain bacterial DNA mostly from the *Proteobacteria phylum*, which represents on average 55% of the reads, followed by *Firmicutes phylum* (24%) and a lower proportion of *Bacteroidetes* (11%) and *Actinobacteria* (8%) phyla without differences due to rumen fluid supplementation. At a deeper taxonomic level, the predominant family was *Oxalobacteracea* followed by *Pseudomonadaceae*, *Lachnospiraceae*, *Ruminococcaceae*, *Sinobacteraceae* and *Enterobacteriaceae* (Fig. 4a). Although *Oxalobacteraceae, Pseudomonadaceae* and *Sinobacteraceae* were also observed in negative blank, their relative abundances were removed from all PBMC samples. Microbial distribution at family level in PBMC and colon content is fully described in supplementary figure 1.

At genus level, the 20 most abundant OTUs in colon and PBMC microbiome were represented in Fig. 4b. Predominant genera found in PBMC were *Pseudomonas, Prevotella, Ruminococcus*, *Sphingomonas* and *Acinetobacter*. Other genera such as *Corynebacterium*, *Butyrivibrio, Bifidobacterium*, *Dorea*, *Coprococcus *or* Blautia,* were also present in both microbial environments. Among the taxa present in higher abundance in almost all PBMC samples, it was observed that several genera (*Acinetobacter, Corynebacterium*, and *Pseudomonas*) containing species that are pathogens with known capacity to infect blood. On the other side, other leading players in the maintenance of the homeostasis in the gut such as *Faecalibacterium prausnitzii* (*Firmicutes phylum*) considered one of the main butyrate producer in the gut and *Lachnospiraceae* members (*Butyrivibrio, Dorea*, *Coprococcus, Blautia and Ruminococcus)* among the main producers of short chain fatty acids, were also observed. In general, less than 8% of OTUs detected were shared between PBMC and colon environment. In this regard, colon content was mainly composed of *Firmicutes* (83%), followed by *Actinobacteria* (8.2%), *Bacteroidetes* (4.8%) and *Proteobacteria* (1.3%).

To further investigate how differences in the microbiome composition could impact the functionality of microbiota, we performed predictive analysis of functional pathways and compared the pathway representation between colon content and PBMC. The mean proportion of predicted KEGG pathways (at level 3) of the metagenome functional content (bacterial genes) and significantly augmented in the PBMC of goats compared to colon content were illustrated in Supplementary Figure 1. Most of them were associated to anti-inflammatory profile of immune cells.

**Fig. 5:**
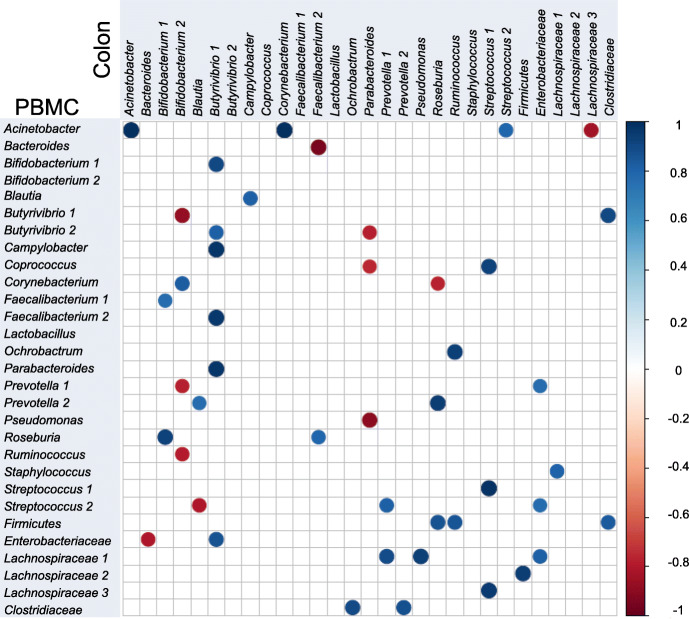
Pearson correlation of microbial community present in colon and PBMC microbiome. Pearson correlation analysis indicated that the variation in bacterial community composition in both environment had significant correlations (*p* = 0.05). The size and intensity of color for each circle represents the strength of the correlation (the larger, darker circles demonstrate a strong correlation), blue colors illustrate positive correlations and red colors illustrate negative correlation coefficient

In order to understand whether the wide range of OTUs found in colon microbial composition were related to circulating microbiota, Pearson correlation analyses were performed. Variation in bacterial community composition detected in colon and PBMC samples showed a significant correlation (Fig. 5). The presence of *Acinetobacter* and *Streptococcus* in colon microbiome correlated (*P* ≤ 0.01) with the same OTU in circulating microbiome. A positive correlation between *Butyrivibrio* in colon and *Bifidobacterium*, *Campylobacter, Faecalibacterium* and *Parabacteroides* in PBMC samples was observed. On the other hand, the presence of *Bifidobacterium* in colon showed a negative correlation with *Butyrivibrio, Prevotella *and* Ruminococcus* in PBMC bacterial environment. *Parabacteroides* also showed a negative correlation with *Butyrivibrio, Coprococcus* and *Pseudomonas*.

## Discussion

Many studies have reported the successful culture and microscopic observation of numerous bacteria from blood of healthy individuals, especially in humans [12]. However, no exhaustive analysis has been performed of the blood microbiome in ruminants and even less in their circulating immune cells. Thanks to the evolution of sequencing technologies and the optimization of the processes, we have successfully characterized the taxonomic profile of the PBMC present in young healthy ruminants in the absence of any pathological condition. As expected, colon gut microbiota was modified by the rumen fluid transplantation, showing different microbial community according to experimental groups. However, its impact in dry matter colon content was not observed. Differences in the colon microbiota could rely on two aspects: i) a change in the microbial colonization pattern in early life with some persistency later in life [37–39] and ii) an indirect effect driven by a higher rumen microbial fermentation of the feed in RLT animals, mostly associated with the presence of rumen protozoa, which could limit the availability of fermentable substrate in the hindgut [38]. In a recent study we have demonstrated that inoculation of young goat kids with rumen fluid from adult goats accelerated the rumen microbial development favouring the presence of rumen protozoa, a higher bacterial diversity and increased abundances of certain bacterial taxa (e.g. Firmicutes and Fibrobacteres) [40]. Our results are in agreement with previous studies [25] where gene expression levels at the rumen epithelium of newborn goats were not affected although feeding management (maternal versus milk replacer) during the first month of life promoted different rumen microbial colonization. In that case, only *Tlr5* expression in rumen varied. In the present study, a higher translocation of bacteria in supplemented goats was suggested as *Tlr5* is expressed mostly on the basolateral side of intestinal epithelial cells for detecting whether bacteria have crossed the gut epithelia. Although, in general it is accepted that bacteria can pass into tissues originating from the gut, in this study, it was revealed that in standard physiological conditions, only a low proportion of microbiota was shared between colon and PBMC. Results in this study suggest non-intestinal sources as main contributors to microbial DNA in circulating PBMC.

Several sources can potentially contribute to add external contamination: bacterial contaminants collected during the sampling in the experimental farm, the manipulation of the samples and the reagents in the laboratory during extraction and sequencing library preparation pipeline could lead to the artificial identification of PBMC microbiome which add a background to the analysis of the blood microbiome and can be misinterpreted as bacteria present in the samples [20, 21, 23, 41]. Hence, in this study negative controls were run all over the process to track potential contamination during the manipulation and sequencing of the samples. In this sense, Poore et al. [8] reported that in silico decontamination did not appear to differentially affect the types of samples under study, validating gold-standard microbiology practices for low biomass studies.

While evidence for the existence of a blood-microbiome in various domesticated mammals and birds do exist [42–44], this study was the first to track the major part of PBMC microbiome in healthy ruminants by *16S rRNA* gene amplicon sequencing. Here, higher abundance of OTUs assigned to *Proteobacteria phylum* (more than 50%) was found in PBMC compared to the colon microbiome (*Firmicutes* more than 80%). In addition, rumen composition also differed from circulating bacterial profile, being *Bacteroidetes* the predominant *phylum* showing significant differences according to treatment, representing 41 and 60% in CTL and RLT groups, respectively (*p* = 0,01) [45]. Relative abundance was followed by *Proteobacteria* and *Firmicutes* although they did not show differences between groups. Other potential source of bacteria to circulating microbiome would be oral cavity as daily activities including chewing or when the barriers between oral environment and the circulatory system are compromised, result in the translocation of oral bacteria into the bloodstream [11, 46]. However, results described in oral secretion did not agree between both ecosystems being the most abundant *phylum* reported *Firmicutes* (50%), followed by *Proteobacteria* (29%), *Actinobacteria* (3.5%) and *Fusobacteria* (1%) [47]. Our results are in agreement with different studies aiming to investigate blood microbiome in healthy human donors where *Proteobacteria* was found over 80%, followed by *Actinobacteria* (between 7 and 10%) and *Firmicutes* (from 3 to 6%) [12–16, 48, 49]. However, the characterization of blood bacterial diversity varies between studies [50]. The abundance of *Proteobacteria* in PBMC comprises both obligate and facultative anaerobic bacteria genera therefore could be related to their ability to tolerate a range of oxic conditions. They are highly present in the gut of neonatal goats [25], specifically in rumen, and other mammals, which are abundant in oxygen immediately *post-partum* contributing to the homeostasis of the anaerobic environment. In general, these results suggest a common circulating bacterial profile on mammals.

Bacterial translocation from gut was described several years ago but mostly related to pathological conditions. However, nowadays more evidence supports bacterial translocation in physiological context. The difference of bacterial profiles between the gut and PBMC could be explained by the role of filter played by the intestinal barrier and immune cells, which limits the translocation of a specific portion of the gut microbiota to the periphery [51–53]. Other compartments of the digestive tract, tissues and organs, such as skin, oral cavity, nasal, and lung mucosa probably are making a significant contribution to the bacterial DNA present in PBMC. It appears that maternal origin would be also accepted. In agreement, Whittle et al. [15] demonstrated that blood-microbiome closely resembles the skin and oral microbiomes and differs substantially from the intestinal microbiome. Due to the low number of animals used in this experiment, it was not sufficient to assert whether PBMC bacterial composition was affected by rumen fluid transplantation. However, results indicated that the bacterial environment inside PBMC is quite stable in time and comprise a core set instead of a dynamic and adaptive group of microorganisms. Probably the specificity and stability of the bacterial DNA is linked to the function that has to perform in such location and only the ones that play a role are the only ones allowed to use PBMC as “vehicles”. As previously mentioned, Poore et al. [8] could discriminate among samples from healthy, cancer-free individuals and those from patients with multiple types of cancer (prostate, lung, and melanoma) using only plasma-derived, cell-free microbial nucleic acids exhibiting a relevant role of blood microbiome in health and disease. Although its biological significance remains to be further explored, the discovery of a PBMC microbiome in ruminants might represent an important step toward a better understanding of the microbial relationships with health.

The information provided here opens the possibility to support different hypothesis in ruminants such as entero-mammary route. However, living, nonviable, or dormant bacteria were not addressed in this study. Therefore, the understanding of the PBMC microbiota will require further investigations in the future. Nevertheless, to provide evidence to the theory of travellers’ microorganism in the blood immune cells, we have recently described the survival capacity of *Akkermansia muciniphila* [54] in healthy human monocytes for more than 3 h.

To conclude, our results demonstrate the presence of a highly diversified PBMC microbiome in healthy ruminants that differs from that in the colon community. The rumen fluid transplantation in early life modified the bacterial community structure in the colon providing a wide range of microbes. However, these microbial differences were not observed in the PBMC. Our results suggest that in healthy physiological conditions, the intestinal barrier plays a critical role limiting the bacterial groups able to reach circulating immune cells. *Proteobacteria* was the most abundant phylum, and *Pseudomonas* and *Prevotella* were the dominant genera in the PBMC microbiome. In this study, a positive correlation between *Butyrivibrio* found in the colon and genera such as *Bifidobacterium* and *Faecalibacterium* detected in PBMC samples was observed. However, this observation does not necessarily implies causality since the origin and role of the physiological PBMC microbiome, and its interaction with the host remains to be elucidated.

## Supplementary Information

**Additional file 1: Supplementary Figure 1.** Microbial distribution at family level in PBMC and colon content in healthy goats.

**Additional file 2: Supplementary Figure 2.** Predicted pathways (level 3) with the most significant differences (*p*-value corrected) augmented in PBMC (orange bars) compared to colon (blue bars) samples.

## Data Availability

Raw sequences were made available at European Nucleotide Archive (ENA) under the project number PRJEB39180.
