# Supplementary figures and images for: Peripheral blood mononuclear cells (PBMC) microbiome is not affected by colon microbiota in healthy goats
Source: Anim Microbiome. 2021 Apr 14;3:28. doi: 10.1186/s42523-021-00091-7 (PMC8048065; doi:10.1186/s42523-021-00091-7)

## Slide 1
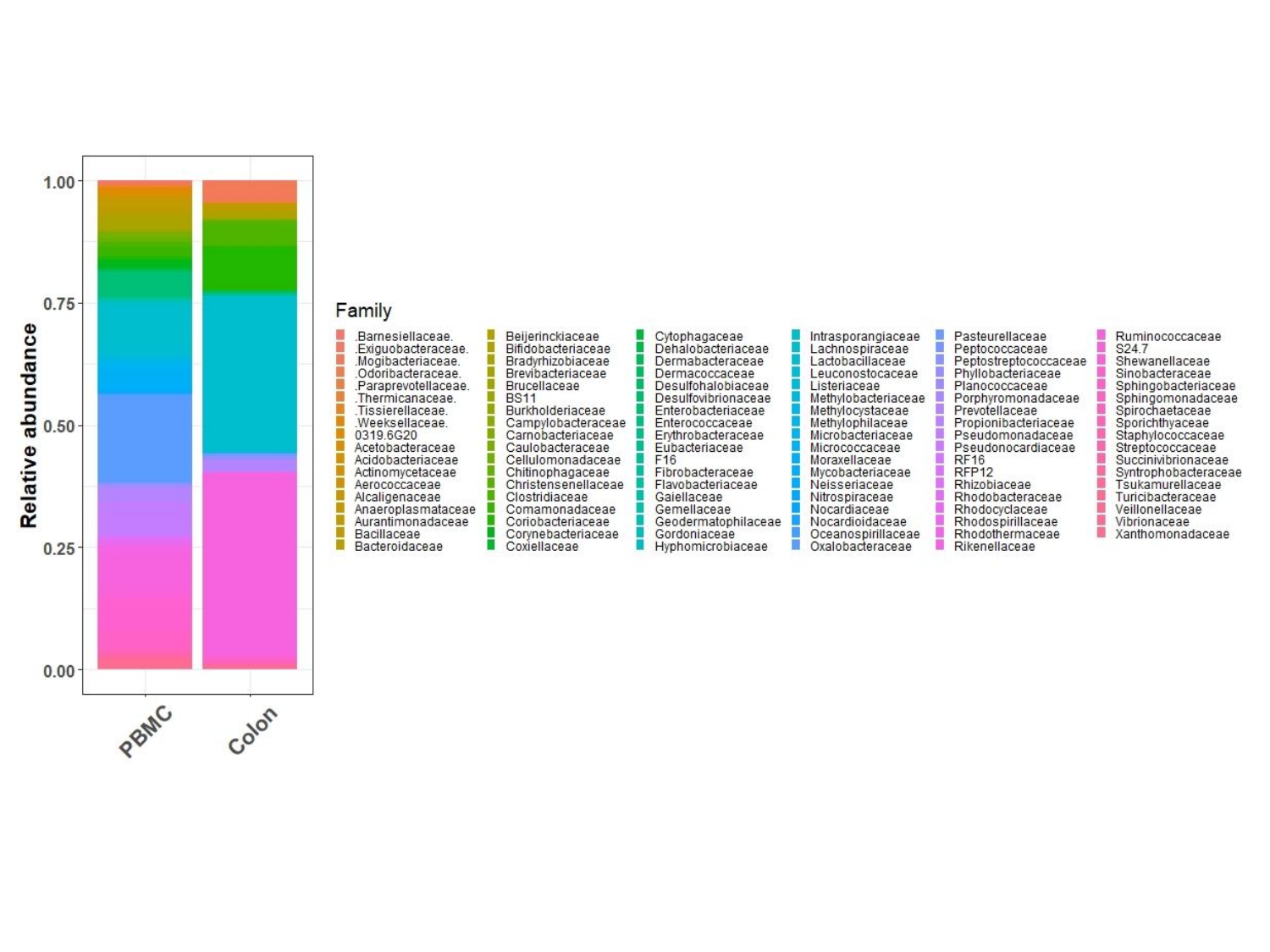

Supplement: Supplementary file 1 — Additional file 1: Supplementary Figure 1. Microbial distribution at family level in PBMC and colon content in healthy goats. [file 42523_2021_91_MOESM1_ESM.pptx]

## Slide 1
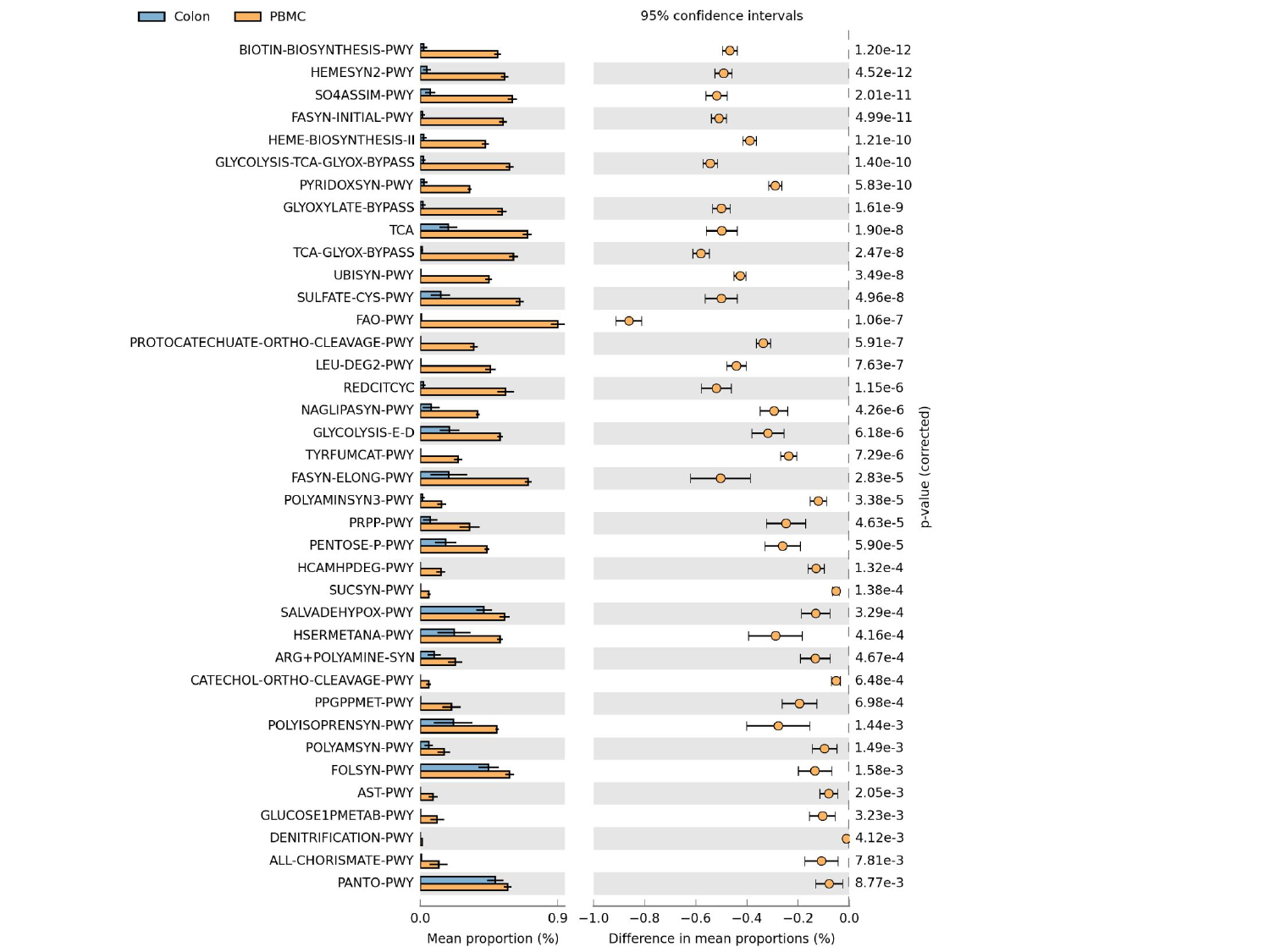

Supplement: Supplementary file 2 — Additional file 2: Supplementary Figure 2. Predicted pathways (level 3) with the most significant differences (p-value corrected) augmented in PBMC (orange bars) compared to colon (blue bars) samples. [file 42523_2021_91_MOESM2_ESM.pptx]
